# Improving the quality of modelling evidence used for tuberculosis policy evaluation

**DOI:** 10.5588/ijtld.18.0660

**Published:** 2019-04-01

**Authors:** N. A. Menzies, C. F. McQuaid, G. B. Gomez, A. Siroka, P. Glaziou, K. Floyd, R. G. White, R. M. G. J. Houben

**Affiliations:** * Department of Global Health and Population, Boston, Massachusetts, USA; † Center for Health Decision Science, Harvard T H Chan School of Public Health, Boston, Massachusetts, USA; ‡ Department of Infectious Disease Epidemiology, London, UK; § TB Modelling Group, TB Centre and Centre for Mathematical Modelling of Infectious Diseases, London, UK; ¶ Department of Global Health and Development, London School of Hygiene & Tropical Medicine, London, UK; # Global TB Programme, World Health Organization, Geneva, Switzerland

**Keywords:** guidance, country-level, resource allocation, mathematical modeling

## Abstract

Mathematical modelling is commonly used to evaluate policy options for tuberculosis (TB) control in high-burden countries. Although major policy and funding decisions are made based on these analyses, there is concern about the variability of results produced using modelled policy analyses. We discuss new guidance for country-level TB policy modelling. The guidance was developed by the TB Modelling and Analysis Consortium in collaboration with the World Health Organization Global TB Programme, with input from a range of TB stakeholders (funders, modelling groups, country TB programme staff and subject matter experts). The guidance describes principles for country-level TB modelling, as well as good practices for operationalising the principles. The principles cover technical concerns such as model design, parameterisation and validation, as well as approaches for incorporating modelling into country-led policy making and budgeting. For modellers, this guidance suggests approaches to improve the quality and relevance of modelling undertaken to support country-level planning. For non-modellers, this guidance describes considerations for engaging modelling technical assistance, contributing to a modelling exercise and reviewing the results of modelled analyses. If routinely adopted, this guidance should improve the reliability, transparency and usefulness of modelling for country-level TB policy making. However, this guidance will not address all challenges facing modelling, and ongoing work is needed to improve the empirical evidence base for TB policy evaluation and develop stronger mechanisms for validating models. Increasing country ownership of the modelling process remains a challenge, requiring sustained engagement and capacity building.

## Abstract (FR)

La modélisation mathématique est habituellement utilisée pour évaluer les options de politiques en matière de lutte contre la tuberculose (TB) dans les pays fortement touchés. Les décisions majeures en matière de politique et de financement sont prises en fonction de ces analyses, mais la variabilité des résultats produits par les analyses de politique modélisées reste préoccupante. Nous discutons d'une nouvelle guidance pour la modélisation de la politique relative à la TB dans chaque pays. La guidance a été développé par le TB Modelling and Analysis Consortium en collaboration avec la programme mondial TB de l'Organisation Mondiale de la Santé, avec la participation d'un range d'acteurs de la TB (financeurs, groupe de modélisation, personnel du programme TB national et experts du domaine). La guidance décrit les principes de la modélisation de la TB dans les pays ainsi que les bonnes pratiques de mise en œuvre des principes. Les principes couvrent les questions techniques comme la conception du modèle, le paramétrage et la validation, ainsi que les approches visant à incorporer la modélisation dans les décisions politiques des pays et le financement. Pour les modélisateurs, cette guidance suggère des approches visant à améliorer la qualité et la pertinence de la modélisation entreprise afin de soutenir la planification au niveau des pays. Pour les non modélisateurs, cette guidance décrit les éléments à envisager pour engager une assistance technique en termes de modélisation, contribuant à un exercice de modélisation, ou à revoir les résultats des analyses modélisées. Si elle est adoptée en routine, cette guidance devrait améliorer la fiabilité, la transparence et l'utilité de la modélisation pour les prises de décisions en matière de TB au niveau des pays. Cette guidance ne résoudra cependant pas tous les défis de la modélisation et un travail continu est requis pour améliorer la base de preuves concrètes afin d'évaluer les politiques en matière de TB et de développer des mécanismes plus solides de validation des modèles. Augmenter l'appropriation par les pays des processus de modélisation reste un défi, exigeant un engagement soutenu et un renforcement des capacités.

## Abstract (ES)

La modelización matemática se suele utilizar con el fin de evaluar las opciones de las políticas de control de la tuberculosis (TB) en los países con alta carga de morbilidad. Las principales decisiones normativas y de financiamiento se adoptan en función de estos análisis; sin embargo, es preocupante observar la variabilidad de los resultados de los análisis modelizados de las políticas. En el presente estudio se examina una nueva orientación en materia de modelización a escala nacional de las políticas contra la TB. El *TB Modelling and Analysis Consortium* elaboró las directrices en colaboración con el Programa Mundial contra la Tuberculosis de la Organización de la Salud, con aportaciones de una diversidad de interesados directos en la TB (financiadores, grupos de modelización, personal de Programas Nacionales contra la TB y expertos en el tema). La orientación describe los principios de la modelización a escala nacional, además de las prácticas adecuadas para ponerlos en práctica. Los principios abordan prioridades técnicas como el diseño del modelo, la parametrización y la validación y también métodos encaminados a incorporar la modelización en la formulación de políticas y la elaboración de presupuestos dirigidas por los países. Estas orientaciones ofrecen a los modelizadores métodos para mejorar la calidad y la pertinencia de la modelización que emprenden con el fin de respaldar la planificación nacional. A otras personas que no participan en la elaboración de modelos, la orientación describe aspectos que se deben tener en cuenta al solicitar asistencia técnica con la modelización, participar en un ejercicio de modelización o evaluar los resultados de los análisis modelizados. Esta orientación, una vez adoptada de manera sistemática, debería mejorar la fiabilidad, la transparencia y la utilidad de la modelización en la formulación de las políticas nacionales en materia de TB. Sin embargo, el documento no aborda todas las dificultades que surgen durante la modelización y se precisan esfuerzos continuos que mejoren la base de datos probatorios para la evaluación de las políticas relacionadas con la TB y la creación de mecanismos más sólidos de validación de los modelos. Lograr una mayor apropiación nacional del proceso de modelización sigue siendo una empresa difícil, que exige un compromiso permanente y fortalecimiento de la capacidad.

MATHEMATICAL MODELLING is commonly used to evaluate disease control options in high-burden settings. In several recent examples, mathematical modelling has been used to guide major policy changes, including revisions to human immunodeficiency virus (HIV) treatment guidelines,^1^,^2^ and South Africa's recent development of an investment framework for tuberculosis (TB) and HIV.^3^–^5^ An increasing number of applications to international TB programme funders now use modelling to justify funding requests, which has been actively promoted by funding and technical agencies. By January 2018, more than 20 low- and middle-income countries had used modelling to inform Global Fund applications, National Strategic Plans or other domestic budgetary or policy processes. Modelling also shapes broader policy discussions,^6^ and there are proposals that modelling evidence be formally considered during World Health Organization (WHO) guideline development.^7^

While models are increasingly used for prospective policy evaluation, it can be difficult for the audience for modelling results (country policy makers, funders) to understand whether modelling results are correct, or how the appropriateness of modelling should be judged.^8^ Moreover, there is increasing concern about the variability of results produced using these analyses, with several recent model comparison exercises demonstrating that different models can produce very different results, even when examining standardised policy questions.^9^–^13^ When model natural history assumptions have been compared against empirical data, these comparisons have shown wide variation between models and systematic deviations from the empirical evidence.^14^,^15^ Ideally, models would be judged by their ability to predict future outcomes,^16^ yet when this predictive validation has been possible, results have been mixed.^17^ In a key example, modelling undertaken before adoption of the Xpert^®^MTB/RIF (Cepheid, Sunnyvale, CA, USA) assay for TB diagnosis in South Africa suggested substantial health impact and attractive cost-effectiveness for Xpert-based diagnostic algorithms.^18^–^20^ However, subsequent pragmatic trials^21^ and an evaluation of South Africa's national Xpert roll-out^22^ demonstrated only a modest impact on diagnosis and no statistically discernable impact on mortality. Subsequent cost-effectiveness analyses showed substantially diminished impact estimates.^23^,^24^

Based on these concerns, a collaboration of international scientific and policy stakeholders has developed principles and good practices for country-level TB modelling. This work was led by the TB Modelling and Analysis Consortium (TB MAC), in collaboration with the WHO Global TB Department, representatives of major funders and technical partners (The Global Fund, the World Bank, the Bill and Melinda Gates Foundation, the US Agency for International Development, the Stop TB Partnership), leading modelling groups, technical experts and country-level TB programme representatives. The guidance is published by the WHO Global Task Force on TB Impact Measurement.

In the present paper, we describe the process of developing the modelling guidance (Methods section) and report on the modelling principles included in this guidance (Results section). In the Discussion, we discuss the role that this modelling guidance can play in the expanding field of country-level TB modelling, identify current challenges for country-level TB modelling and propose additional actions that could improve the rigour and usefulness of modelling to support TB policy making.

## METHODS

### Scope

The guidance was developed 1) to describe the appropriate use of mathematical models to support national TB policy and planning, including applications to international funding agencies, 2) to consider both epidemiological and economic aspects of modelling, to address all considerations for evaluating competing policy options (e.g., projecting future epidemiological outcomes, cost estimation, analyses of cost-effectiveness and allocative efficiency), and 3) to consider the technical aspects of modelling, as well as the approaches used to apply modelling in a given country, and integrate modelling evidence into decision-making. The guidance was developed to be read in conjunction with other relevant guidance documents, such as the Gates Reference Case for Economic Evaluation,^25^ and additional criteria prescribed by funders or other stakeholders.

### Target audience

The target audience is the participants and stakeholders in country-level TB modelling. This includes individuals who build and/or apply models, policy makers, technical experts, international funding and technical partners, and other individuals and organisations that support TB policy making. For modellers, the guidance suggests approaches to improve the quality, relevance, transparency and timeliness of modelling. For non-modellers, this guidance describes considerations when engaging modelling technical assistance, contributing to a modelling exercise or reviewing the results of modelled analyses.

### Development process

An initial outline was prepared by a small writing committee and reviewed by 30 expert stakeholders, including TB modellers, country stakeholders, donors and advocates. Suggestions were incorporated and a full draft of the guidance developed. Following a further round of review, the draft guidance was presented at the TB MAC annual meeting in Glion, Switzerland (18–22 September 2017), where input was invited from a wider stakeholder group that included modelling groups, international stakeholders and funders and other technical experts. Further input was provided after this meeting by country stakeholders and technical experts, and a final draft reviewed and endorsed by the WHO Global Task Force on TB Impact Measurement in May 2018.

### Format

The guidance is organised as 10 principles to guide country-level TB modelling. These principles were designed to be general enough to apply to the majority of scenarios that arise in a modelling application. For each principle, a number of ‘good practices’ were developed. While unlikely to apply to all situations, these practices suggest concrete actions for operationalising each principle.

### Role of the funding source

Employees of the funder participated during guidance development discussions. The funder had no role in manuscript development or submission decisions.

## RESULTS

The 10 principles for country-level TB modelling are described below. These principles were endorsed by the WHO Global Taskforce on TB Impact Measurement, and included the published guidance,^26^ which can be found at http://www.who.int/tb/publications/2018/country_modelling/en/.

Table 1 provides a summary description, and the Figure shows how these principles relate to the steps of a typical modelling application. Good practices for each principle are provided in the supplement, and the guidance document also provides examples from country-modelling applications indicating when and how the principles would apply.

**Figure:**
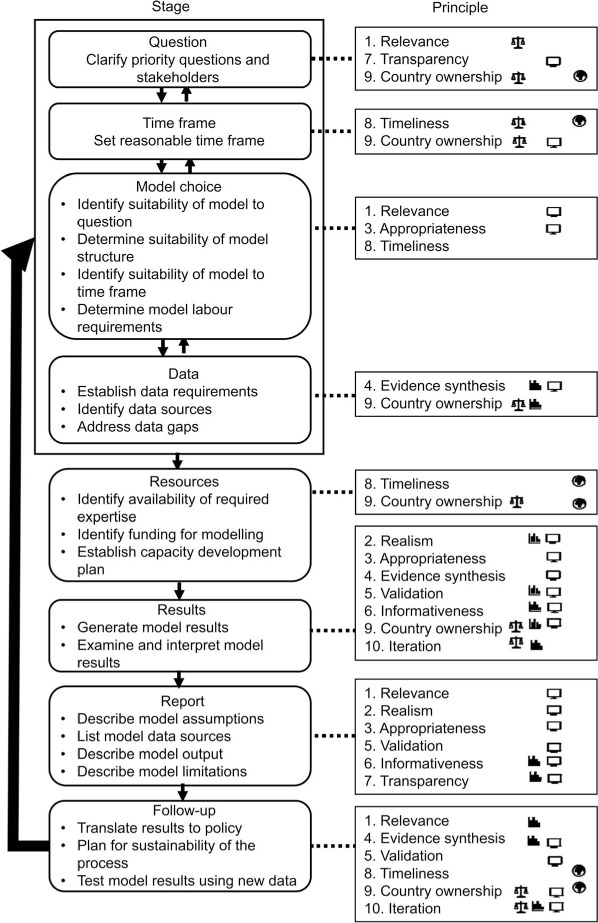
Flow chart of steps involved in a typical modelling project*. * Numbers refer to principles described in the text. Icons shown on right of the figure describe suggested lead actors for each step in the modelling process, i.e., the individual or group primarily responsible for implementing principles from a given practice. Actors include in-country decision makers ( 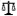), in-country experts ( 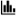), modellers ( 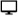) and international funders ( 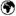). Other actors may also have a role in contributing towards activities or in creating the demand for them.

**Table 1:** Principles for country-level TB modelling

1	Relevance: modelling should assess the policies and outcomes relevant to the decision-maker
2	Realism: modelling should explicitly consider implementation challenges that may reduce the effectiveness or increase the costs of interventions when introduced into routine practice, and examine the plausibility of assumptions required for policy success
3	Appropriateness of model structure: the model design should be justified in terms of the questions and local context being considered—the structure should be sufficiently detailed to represent the mechanisms generating outcomes, but avoid unnecessary complexity
4	Consideration of all evidence: modelling should consider all available evidence relevant to the decision problem
5	Validation: where possible, model results should be compared with evidence not used for model parameterisation or calibration to understand the consistency of modelling results with other evidence
6	Informativeness: modelled analyses should report a rich set of results describing consequences for a range of outputs and outcomes to provide a deeper understanding of the scenarios being modelled and model functioning
7	Transparency: modelling results should be accompanied by a clear description of the evidence that supports the main findings, limitations of the modelling approach, uncertainty in modelled estimates and the sensitivity of results to different assumptions. Conflicts of interest should be avoided if possible, or otherwise described explicitly
8	Timeliness: modelling activities should be organised to provide results at the time they are required for decision-making
9	Country ownership: modelling should be conducted with the full participation of local stakeholders at each stage of the process
10	Iteration: modelling should involve an iterative process of engagement and be reconsidered in the light of new evidence

### Principle 1: relevance

Modelling should assess the policies and outcomes relevant to the decision maker. Without good collaboration between the users and producers of modelling information, it is all too easy for modelling to evaluate strategies that differ from those being considered by decision makers. Where a pre-existing model is used for a new planning exercise, the model may be insufficiently tailored to the local context or strategies may be excluded from consideration because they are not already built into the model. Ideally, the modelling exercise can play a beneficial role in shaping the policy scenarios being considered and identifying outcomes of interest, with the specificity required to parameterise a model forcing participants to think through the details of how a policy or new intervention would be implemented, the mechanisms through which it would impact costs and health outcomes, and how these should be summarised to inform the decision.

### Principle 2: realism

Modelling should explicitly consider implementation challenges that may reduce policy effectiveness or increase policy costs, and examine the plausibility of assumptions required for policy success. Modelling is often called upon to evaluate novel interventions or proposals to expand the coverage or quality of routine services above current levels. For these scenarios, there is generally less information about their effects and costs in typical programmatic settings. This can produce overly optimistic projections of the effectiveness or cost-effectiveness of proposed policies, particularly when initial evidence is obtained in high-capacity clinical settings or for subpopulations where effect sizes are larger, or when there is external pressure for modelling to ‘be ambitious’. Historical experience is likely to be the best starting point for modelling assumptions about the pace and success of implementation.

### Principle 3: appropriateness of model structure

The model design should be justified in terms of the questions and context being considered—the structure should be sufficiently detailed to represent the mechanisms generating outcomes, but avoid unnecessary complexity. Model development commonly requires many decisions about model structure, dealing with how to represent the population affected by a particular policy and how to describe their transition through demographic and epidemiological processes and the receipt of health care. These choices balance the conflicting priorities of 1) faithfully representing the process being modelled, which commonly leads to more detailed modelling approaches (e.g., greater heterogeneity of the modelled population or more complicated functions describing health state transitions), and 2) developing a model whose processes are transparent and understandable for both the modeller and the modelling audience, which may be facilitated by simpler modelling approaches. While there will be some structural choices that promote both of these aims simultaneously, most choices involve a trade-off between realism and parsimony. Moreover, the decision to add more detail on a particular part of the model will not necessarily improve the validity of the results—as model complexity increases, it can become more difficult to explain unexpected results and identify errors. Similarly, given the constrained resources and short timeframe commonly available for modelling applications, if the model runs slowly and requires substantial computing resources, this can limit the opportunities to fully investigate parameter uncertainty, assess different scenarios and iteratively improve the analysis through feedback from modelling stakeholders.

### Principle 4: consideration of all evidence

Modelling should consider all available evidence relevant to the decision problem. Mathematical models specify a sequence of relationships linking the actions described under a policy scenario to the health and economic outcomes of interest. Typically, many different evidence sources are required to parameterise these relationships, and errors in any of these parameters could affect results. While there will be finite time and resources available to collate inputs, all key data and evidence should be identified and incorporated to produce valid and accurate results. Where there is substantial uncertainty in key parameter inputs, this uncertainty should be thoroughly investigated, and the implications reported alongside the main analytic results. Adjusting parameter values so that model predictions are consistent with observed data (model calibration) can improve the validity of future projections and increase confidence among consumers in modelling results. However, calibration should be undertaken carefully to avoid over-fitting and acknowledge potential biases in calibration data.

### Principle 5: validation

Where possible, model results should be compared with evidence not used for model parameterisation or calibration to understand the consistency of modelling results with other evidence. Given the complexity and number of assumptions involved in mathematical models, it is difficult to confirm that the model will produce valid results by scrutinising model inputs and structure. Further evidence that a model is reasonable can be gained by comparing model outputs to external estimates for these outputs. Sources of these estimates could include the results of empirical studies, similar modelling efforts or the experience of subject matter experts. None of these comparisons can guarantee that the results of an analysis are valid, but instead provide confirmation that some aspects of model predictions are consistent with external data, or alternately reveal conflicts for further investigation.

### Principle 6: informativeness

Modelled analyses should report a rich set of results describing a range of outputs and outcomes to provide a deeper understanding of policy scenarios and model functioning.

TB policy or planning options will likely have implications for a range of different outcomes of interest to decision makers. For example, an intervention that improves the quality of TB care in marginalised communities will most immediately reduce morbidity and mortality in individuals with active TB disease, but could also have implications for Mycobacterium tuberculosis transmission and TB incidence, trends in TB drug resistance and the socioeconomic distribution of TB burden. In conventional economic evaluation, these various outcomes are combined in a single measure of health benefit—such as disability-adjusted life-years (DALYs) averted or deaths averted—to summarise the overall health implications of a policy. Calculating a single summary metric facilitates the process of identifying optimal choices (e.g., policies that maximise health benefits for a given budget envelope).

Nonetheless, summary measures may not capture all health outcomes of interest to decision makers. For example, although total DALYs or deaths averted will not describe the distribution of health benefits across the population, such distributional information is relevant if reducing inequality is a policy goal. Reporting results for multiple outputs and outcomes can provide a more complete description of policy consequences. Moreover, providing a rich set of results can help decision makers develop a deeper understanding of how interventions work, how different outcomes relate to each other and the timing of effects. Similar considerations apply to resource needs estimates. While an estimate of total resource needs is often required, it is also useful to provide cost estimates disaggregated according to when resources are needed, what budget they draw on and how they compare with existing expenditures. Finally, reporting a more informative set of results can facilitate additional reality checks, allowing modelling participants to confirm that anticipated programme changes are plausible.

### Principle 7: transparency

Modelling results should be accompanied by a description of the evidence that supports main findings, limitations of the modelling approach, uncertainty in modelled estimates and the sensitivity of results to different assumptions. Conflicts of interest should be avoided if possible, or otherwise described explicitly. Models typically reach a level of complexity that makes their mechanisms difficult to understand for anyone lacking the necessary time to read and review extensive documentation, particularly stakeholders who are less familiar with modelling methods. Modelling commonly requires assumptions that have only weak empirical support, yet due to the sheer number of assumptions being made it is difficult for a consumer of modelling results to know which assumptions are important and which have only a minor influence on the outcomes of interest. Nevertheless, it is critical that consumers of modelling results have the information available to understand the strengths and limitations of modelling results, and main threats to validity.

The fact that modelled analyses are complicated and subject to many analytic decisions means that conflicts of interest can be particularly problematic. Conflicts may arise where there are significant commercial, professional, political or other interests involved in a particular decision. If analytic approaches are chosen to favour a preferred outcome, this may not be apparent to a non-expert audience, or even an expert audience aware of the range of possible modelling approaches. For this reason, important conflicts of interest among the participants in a modelling process should be identified and avoided where possible. It may not always be possible to avoid conflicts, in which case an explicit statement describing the conflicts should accompany the results.

### Principle 8: timeliness

Modelling activities should be organised to provide results at the time they are required for decision-making. Ideally, this principle would not conflict with the other principles. However, in practice the need to produce results quickly can reduce the opportunities to test all aspects of a model, and can reduce the time and opportunities for stakeholders to review results, raise questions and refine scenarios. Allowing for a sufficient lead-in time in the policy planning process should help alleviate potential conflicts between this and the other principles.

### Principle 9: country ownership

Modelling should be conducted with the full participation of local stakeholders at each stage of the process. Modelling is more likely to be useful when conducted with the full participation of relevant stakeholders. Their involvement means that modelling assumptions and modelled scenarios are more likely to be appropriate, and that results are fully understood and considered by policy makers when making decisions. Country ownership is not guaranteed in situations where modelling is conducted by external technical experts, and where the need for format of the modelling exercise are driven by external funding agencies. In these situations, greater effort may be needed to fully engage important stakeholders. In any country, there will be existing initiatives for the collection and use of data to inform programme planning. Coordination with these efforts will improve the quality of data available for modelling, and reduce the chance that decision makers receive conflicting policy advice.

### Principle 10: iteration

Modelling should involve an iterative process of engagement, and be reconsidered in the light of new evidence. Given the complexity of modelling and decision-making, the process of identifying candidate policies or interventions, and the evidence to describe them, is likely to be iterative. It is important that the modelling approach allows for this iteration between adaptation of the model and evaluation of results. After a modelling exercise is complete, the results should remain open to criticism and revision in light of new evidence, and a clear way forward to improving the process should be identified.

## DISCUSSION

The use of mathematical modelling to support TB policy making has been encouraged by major funders and adopted by several high-burden countries. These quantitative planning exercises can provide evidence on proposed interventions, support funding applications and improve the impact of limited resources. The country-level TB modelling guidance was developed to provide a framework—as well as pragmatic advice—for how TB modelling and related technical assistance can support country decision-making.^26^ The principles described in this guidance cover the design and estimation of the mathematical models themselves, as well as methods for identifying and synthesising evidence, and approaches for incorporating modelling into country decision-making. These principles serve three higher-level goals: 1) that model-informed policy evaluation makes the best use of available evidence, 2) that modelling is incorporated into policy making in a way that clearly recognises the strengths and weaknesses of modelled estimates, and 3) that modelling supports (rather than replaces) policy making as a deliberative, country-led process.

The process of developing this guidance revealed several challenges facing country-level TB modelling (Table 2). A number of these challenges can be addressed in the context of an individual modelling application, and are addressed in the guidance. For example, the guidance speaks clearly on the need to anticipate implementation challenges that could reduce effectiveness or increase costs for novel policies to achieve more credible projections. The guidance is also clear on the need to transparently describe the evidence base supporting a policy projection and how weaknesses in this evidence could affect policy recommendations, so that users can weigh the robustness of policy decisions based on the modelling results.

**Table 2:** Challenges for country-level TB modelling identified during guidance development process

1	Limitations in the data and evidence available to inform modelled analyses
2	Limitations in the ability of models to represent complex policy scenarios, such as targeting of risk groups not represented in existing models
3	Difficulty in anticipating factors that could negatively impact the outcomes of modelled policy scenarios, such as those that involve novel interventions or aggressive expansion of existing services
4	Difficulty in describing the uncertainty in modelled results and how this should impact decision-making
5	Differences in the modelling and estimation approaches taken by modelling teams, with the potential that different models could provide different policy advice, given the same country context and policy question
6	Scarcity of human resources (worldwide and within high-burden countries) to meet the demand for modelling technical assistance, and lack of information for country TB programmes on what modelling support is available
7	Differences in the level of experience, understanding or expectations of the modelling process by in-country stakeholders and international funders, and related to this, difference in the confidence placed in modelled analyses by local and international stakeholders

Other challenges will be difficult to resolve in an individual application, but are amenable to ongoing changes in the practice of modelling. The difficulty of adapting models to reflect individual country settings and policy preferences is a consequence of the current modelling paradigm, where a modelling application typically involves an international technical assistance provider tailoring a generic model to answer country-specific policy questions.^6^ This process often occurs over a short period of time and can require approximations that might be reasonable, given the imperative to answer urgent policy questions, but that still leaves room for improvement. As modelling becomes more commonplace, country needs are likely to be better understood before an application begins, reducing the need for short-term approximations.

The natural progression of this is institutionalisation of modelling within countries, as is happening in South Africa.^5^ Such institutionalisation requires sustained investments in capacity building. As a result of these efforts, models are better prepared to answer policy questions when needed, and decision makers better understand how to interpret modelling evidence. Such institutionalisation may not be possible for all countries, but repeated exposure of decision makers to modelling evidence—and repeated exposure of modellers to decision makers and new policy questions—will mean that all participants in a modelling application start the application better prepared and become more thoughtful consumers of the results of these applications.

Another challenge is the difficulty of validating models. In almost all cases, the modelling results used for decision-making, such as projections of long-term outcomes, and incremental cost-effectiveness ratios, cannot be validated directly, as the empirical information to do so may never be available. Moreover, as many threats to validity will be specific to an individual setting and policy question, a model cannot itself be considered ‘valid’ separate from the details of how it is used in an individual application. These facts provide a partial explanation for the variable results produced by model comparison exercises. However, intermediate outcomes, such as descriptions of current epidemiology and programme functioning, are more amenable to validation. Future efforts to facilitate the validation of intermediate outcomes could strengthen the quality of modelling evidence and reduce variation that is inconsistent with available empirical evidence. Even for outcomes where empirical information is weak, such as the prevalence of latent tuberculous infection, the force of infection and the proportion of incident disease due to recent infection, routine collection of estimates generated during modelling exercises could provide broader insight into model functioning, and explain divergent modelling results. While requiring substantial time and effort, model comparison exercises are invaluable for investigating where and why models differ, and facilitating knowledge sharing between modelling groups.

One challenge—weaknesses in the evidence used for modelling—may require action beyond the traditional participants in policy modelling. While the evidence for modelling will always be imperfect, the process of developing this guidance identified several critical evidence gaps. Central among these was evidence on the effectiveness of approaches to improve the coverage and quality of existing TB interventions. These incremental programme improvements are less likely to be considered in formal trials, but are central to efforts to strengthen TB control. For current modelling applications, this evidence gap is commonly filled by expert opinion. While this may be a practical solution given the time constraints of a typical modelling application, it introduces uncertainty and subjectivity for a key determinant of policy impact. Modelling would benefit from additional efforts to collect and synthesise empirical data on policy costs and effects, and understand health system and programmatic constraints.^27^ During the development of this guidance, there was discussion about whether modellers should ever refuse to report results for policy scenarios where evidence is weak. It was decided not to include this in the guidance, but the incentives for modellers to report their best estimates for the questions posed to them, and the potential for policy makers to make decisions based on point estimates reported from these analyses, means that data limitations may be difficult to identify when reviewing modelling results, and thus underappreciated. Even where uncertainty is reported, methods are not well developed for supporting decision makers to act on this information.

## CONCLUSION

Modelling is an unavoidable consequence of the desire to understand the future impact of policy choices. Predicting policy outcomes can be accomplished by using a formal mathematical model, by direct extrapolation from local empirical studies, generalising from similar programmes or countries or by relying on expert opinion. Each of these approaches requires assumptions that should be evaluated critically. None of these approaches can dispel the epistemic uncertainty inherent in policy making, but a desirable approach will transparently communicate the uncertainties associated with analytic results, and demonstrate their implications for decision-making. Transparency about the uncertainty in modelling results will also incentivise efforts to collect and synthesise empirical data on policy costs and effects, and to understand how health system and programmatic constraints should be reflected in modelled analyses.^24^

If adopted, the guidance we have described will improve the quality and utility of modelling. To support this process, TB MAC and modelling stakeholders are developing tools and processes to facilitate the implementation of the guidance for routine modelling work. This involves the development of quantitative benchmarks for model validation, standardised reporting formats to demonstrate good practices were followed and a mechanism to provide independent expert review of modelling applications. Taken together, these benchmarking, reporting and reviewing activities will provide tools to reveal where a given modelling application is inconsistent with existing evidence or best-practice modelling approaches. If routinely applied, these tools could strengthen the incentives for high-quality modelling work, and tighten the link between modelling results and the evidence used to justify them, supporting the ongoing improvement of modelling as an aid for country-level TB decision-making.
